# Biginelli Reaction Synthesis of Novel Multitarget-Directed Ligands with Ca^2+^ Channel Blocking Ability, Cholinesterase Inhibition, Antioxidant Capacity, and Nrf2 Activation

**DOI:** 10.3390/molecules28010071

**Published:** 2022-12-22

**Authors:** Rim Malek, Alexey Simakov, Audrey Davis, Maciej Maj, Paul J. Bernard, Artur Wnorowski, Helene Martin, José Marco-Contelles, Fakher Chabchoub, Patrick Dallemagne, Christophe Rochais, Krzysztof Jozwiak, Lhassane Ismaili

**Affiliations:** 1 Laboratoire LINC UR 481, Pôle de Chimie Médicinale, University Franche-Comté, UFR Santé, 19, rue Ambroise Paré, F-25000 Besançon, France; 2 Laboratory of Applied Chemistry: Heterocycles, Lipids and Polymers, Faculty of Sciences of Sfax, University of Sfax, Sfax 3000, Tunisia; 3 PEPITE EA4267, University Franche-Comté, F-25000 Besançon, France; 4 Centre d’Etudes et de Recherche sur le Médicament de Normandie, Normandie University, Unicaen, CERMN, 14000 Caen, France; 5 Department of Biopharmacy, Medical University of Lublin, ul. W. Chodzki 4a, 20-093 Lublin, Poland; 6 Laboratory of Medicinal Chemistry (IQOG, CSIC), C/ Juan de la Cierva 3, 28006 Madrid, Spain

**Keywords:** Biginelli reaction, multitarget-directed ligands, calcium channel antagonism, Nrf2

## Abstract

Novel multitarget-directed ligands **BIGI 4a-d** and **BIGI 5a-d** were designed and synthesized with a simple and cost-efficient procedure via a one-pot three-component Biginelli reaction targeting acetyl-/butyrylcholinesterases inhibition, calcium channel antagonism, and antioxidant ability. Among these multitarget-directed ligands, **BIGI 4b**, **BIGI 4d,** and **BIGI 5b** were identified as promising new hit compounds showing in vitro balanced activities toward the recognized AD targets. In addition, these compounds showed suitable physicochemical properties and a good druglikeness score predicted by Data Warrior software.

## 1. Introduction

Alzheimer’s disease (AD) is considered an extensively complex and multifactorial neurodegenerative disorder. AD is the leading cause of neurocognitive disorders and presents a high social and economic cost estimated at USD 604 billion worldwide [1]. Despite the large number of scientific publications (196K in PubMed), the available drugs are limited to a temporary relief of symptoms [2], while specific and effective treatments for this disease are struggling to reach the market.

Indeed, AD has a complex physiopathology including various biological phenomena and highly interconnected pathological mechanisms that are hallmarked by: (i) neuronal death leading to dysfunction of the acetylcholine (ACh) receptor system in neurons; (ii) extracellular deposits of amyloid-beta (Aβ) peptide resulting from the accumulation of abnormal levels of its insoluble aggregates; (iii) the presence of hyperphosphorylated tau proteins, causing the formation of neurofibrillary tangles; (iv) neuroinflammation induced by high concentrations of pro-inflammatory cytokines released by activated microglia and astrocytes; and (v) disrupted homeostasis of mitochondria and homeostasis of biometals (Cu, Fe, and Zn) related to their contribution to Aβ peptide aggregation, or hydrogen peroxide (H_2_O_2_) resulting from monoamine-oxidase-catalyzed deamination of biogenic amines such as adrenaline, dopamine, and serotonin [3] irreversibly leading to oxidative stress (OS).

Faced with this pathophysiological complexity, the multitarget strategy initially introduced by Melchiorre and colleagues [4] based on the development of new ligands able to bind simultaneously to various enzymatic systems or receptors involved in the progress of AD seems to be the most appropriate strategy to find new effective drugs. 

Accordingly, by following this paradigm several multitarget-directed ligands for AD were developed with promising profiles by many research groups [5,6,7,8,9]. Our contributions in this area to developing novel multitarget-directed ligands are based on the use of multicomponent reactions (MCRs) for their easy performance, time saving, versatility, and the diversity of the resulting scaffolds [10,11,12,13]. In addition, MCRs are atom economic as most atoms of the reactants, if not all, are found in the product, [14] and therefore respond to the challenges of sustainability (“green chemistry”) [15].

**Figure 1:**
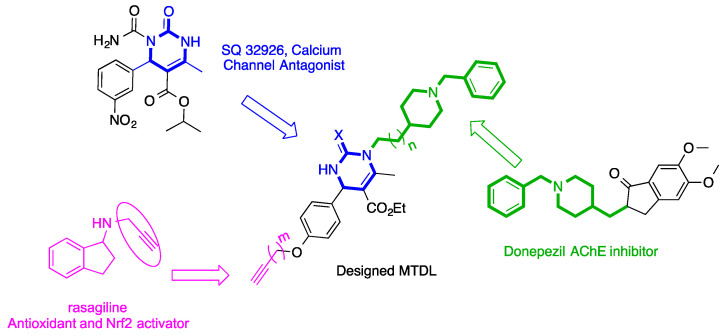
Design of multitarget-directed ligands BIGI **4a-d** and BIGI **5a-d**.

Continuing with our contributions in this area, we report here the design, synthesis via Biginelli MCR, and biological evaluation of a new family of **BIGI 4a-d** and **BIGI 5a-d** as new multitarget-directed ligands resulting from the combination of three scaffolds of interest for AD: (i) a typical Acetylcholinesterase (AChE) inhibitor motif, such as the benzylpiperidine present in donepezil; (ii) the central dihydropyrimidone core which has a potential calcium channel modulation activity similar to classical dihydropyridines [16], such as SQ32926 [16,17]; and (iii) propargyl ether residue, an electrophilic substrate, as an analogue of propargyl amine which is known as an antioxidant able to direct scavenge ROS/RNS [18,19,20] and present in selegiline and rasagiline, both able to induce nuclear translation of nuclear factor erythroid 2-related factor (Nrf2) and increase binding to the antioxidant response element (ARE) [21] (Figure 1).

Indeed, AD is associated with low levels of ACh. This dysfunction can be reversed by the use of cholinesterase (ChE) inhibitors, one of the main treatment options available [22]. The cytosolic calcium overload leads to mitochondrial damage and activation of cell apoptosis [23] and has been identified as a crucial factor in AD. Consequently, calcium channel blockade is commonly recognized as a useful pharmacological tool in the treatment of AD. Antioxidants and Nrf2 pathway induction in the AREc32 cell line are also interesting pharmacological targets for the development of new drug candidates. The Keap1-Nrf2-ARE signaling pathway constitutes one of the most important endogenous antioxidant mechanisms able to regulate the production of oxidants. This is based on the activation of nuclear factor (erythroid-derived 2)-like 2 (Nrf2) [24] which is a very important protein in the organization of antioxidant defenses by triggering the endogenous expression of detoxifying enzymes and leads to the downregulation of iNOS and COX-2 enzymes.

**Scheme 1:**
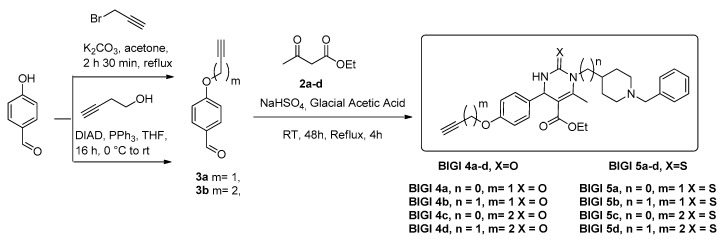
Synthesis of targeted multitarget-directed ligands BIGI **4a-d** and BIGI **5a-d** using one-pot Biginelli reaction.

Thus, the new multitarget-directed ligands BIGI **4a-d** and BIGI **5a-d** (Scheme 1, Table 1) were investigated for their calcium channels, ChE inhibition, antioxidant power, and Nrf2 activation. From these studies, we identified BIGI **4b,** BIGI **4d**, and BIGI **5b** as new and very promising hit agents for potential AD therapy combining activities against three biological targets, as these compounds are good Ca^2+^ channel blockers with potent cholinesterase inhibition and an Nrf2-ARE-activating effect.

## 2. Results

### 2.1. Synthesis 

**Scheme 2:**
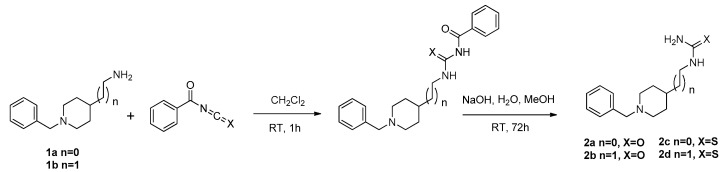
Synthesis of ureas and thioureas derivatives **2a-d**.

The synthesis of the new multitarget-directed ligands BIGI **4a**-**d** and BIGI **5a-d** was carried out in a one-pot Biginelli reaction of aldehydes **3a**-**b** with ethyl acetoacetate and ureas **2a-d** using sodium bisulfate as a catalyst in acetic acid at room temperature for 48 h and refluxed for an additional 4h. (Scheme 2). Aldehyde **3a** was prepared from the 4-hydroxybenzaldehyde and propargyl bromide under typical Williamson reaction conditions (Scheme 1), whereas aldehyde **3b** was prepared by the Mitsunobu reaction, under the conditions described by Mertens [25], from but-3-yn-1-ol and 3-substituted 4-hydroxybenzaldehydes, in the presence of PPh_3_ and di-isopropyl azodicarboxylate (DIAD), in THF at room temperature (rt) (Scheme 1). Ureas **2a-d** were obtained from commercial benzylpiperidines **1a-b** (*n* = 0,1) with benzoyl isocyanate or benzoylthioisocyante, in CH_2_Cl_2_ at ambient temperature for 1 h, followed by hydrolysis of the resulting compounds with NaOH for 72 h [26] (Scheme 2). All new compounds were characterized by ^1^H and ^13^C NMR and ESI-MS showing data in good agreement with their structure which are collected in the Experimental Section and Supplementary Information. 

### 2.2. Biological Evaluation

To verify the effectiveness of our compounds to simultaneously hit the selected targets, compounds BIGI **4a-d** and BIGI **5a-d** were submitted to inhibition of human ChE (hChE) and blockade of the calcium channel as well as antioxidant activity and Nrf2 transcriptional activation. 

#### 2.2.1. Inhibition of hAChE and eqBuChE 

For the ChE inhibition experiments, we used the Ellman protocol [27] with *h*AChE and eqBuChE, and Donepezil and Tacrine were used as references. As shown in Table 1, four compounds, BIGI **4b**, BIGI **4d**, BIGI **5b**, and BIGI **5d**, showed good *h*AchE inhibition with IC_50_ equal to 342, 462, 352, and 1271nM, respectively, compared with Donepezil which showed an IC_50_ equal to 12.7 nM. The two best compounds, BIGI **4b** and BIGI **5b**, are only 27-fold less active than Donepezil.

For the structure–activity relationship (SAR), the linker length between benzylpiperidine and the central dihydropyrimidine nitrogen atom seems to play an important role. Indeed, no activity was observed when the linker length was *n* = 0 while all compounds with *n* = 1 were AChE inhibitors. 

Regarding the length of the linker between the oxygen and the triple bond, a slight variation in activity was observed between BIGI **4b** (m = 1, 342 nM) and BIGI **4d** (m = 2, 462 nM), both carrying a dihydropyridinone. However, a strong variation was observed between the analogues bearing dihydropyrimidinethiones BIGI **5b**, (m = 1, IC_50_ = 352 nM) and BIGI **5d** (m = 2, IC_50_ = 1271 nM), suggesting an effect of the sulfur atom on the activity. 

Regarding *eq*BuChE inhibition, except for compound BIGI **4c**, all compounds showed micromolar inhibition ranging from 4.78 μM for BIGI **4b** to 33.8 μM for BIGI **5c** compared with tacrine which showed an IC_50_ equal to 2.2 nM. Interestingly, the two best AChE inhibitors, BIGI **4b** and BIGI **5b**, are also the best BuChE inhibitors with IC_50_ equal to 4.78 μM and 5.43 μM, respectively.

For SAR, the compounds with linker length *n* = 1 always showed better activity than their analogues with *n* = 0.

**Ca^+2^ channel blockade.**

The Ca^+2^ channel blockade capacity of compounds BIGI **4a-d** and BIGI **5a-d**, and nimodipine as standard, at 10 μM concentration was carried out following the usual methodology [28]. As shown in Table 1, the observed % values ranged from 40 (BIGI **4c**) to 74 (BIGI **5b**). Seven of the eight compounds showed better calcium inhibition than the standard nimodipine. The most potent compounds were, in increasing order, BIGI **5c** (63%), BIGI **4b** (67%), BIGI **5d** (68%), and BIGI **5b** (74%), thus comparing very favorably with nimodipine (50%). From the point of view of the structure–activity relationship (SAR), compounds bearing *n* = 1 length as the linker always showed better results than those bearing *n* = 0. For the same lengths of the linkers m and *n*, the compounds with thioureas were always more active than the analogues with urea moiety.

**Antioxidant assay.**

The antioxidant activity of compounds BIGI **4a-d** and BIGI **5a-d** was determined by the ORAC-FL method. [29] Their radical scavenging properties are expressed as Trolox equivalents (TE) units. Melatonin was used as the positive control with an ORAC value of 2.45 [30]. As shown in Table 1, all compounds showed good antioxidant capacity. The best compounds were, in ascending order, BIGI **4b** (1.60 TE), BIGI **5c** (1.75 TE), and BIGI **4d** (1.85 TE) and were found to be on average only 1.4 times less active than melatonin. Concerning the structure–activity relationship, no clear SAR could be established.

**Table 1:** Cholinesterases inhibition, calcium blockade percentages, and ORAC (TE) values for compounds BIGI **4a-d** and BIGI **5a-d** compared with reference compounds.

Compounds	*h*AChE IC_50_ (nM) ± SD ^a^	*eq*BuChE IC_50_ (μM) ± SD ^a^	Calcium Antagonism (% Inhibition at 10 μM) ± SD	ORAC ^b^
**BIGI** **4a**	-	31.2 ± 1.70	45 ± 11	1.44 ± 0.06
**BIGI** **4b**	342 ± 64	4.78 ± 0.58	67 ± 10	1.60 ± 0.15
**BIGI** **4c**	-	-	40± 13	0.98 ± 0.03
**BIGI** **4d**	462 ± 40	6.63 ± 0.71	50 ± 13	1.85 ± 0.16
**BIGI** **5a**	-	7.64 ± 0.79	46 ± 18	1.35 ± 0.04
**BIGI** **5b**	352 ± 15	5.43 ± 0.24	74 ± 16	1.41 ± 0.06
**BIGI** **5c**	-	33.8 ± 7.89	63 ± 18	1.75 ± 0.06
**BIGI** **5d**	1271 ± 5	8.54 ± 0.71	68 ± 12	1.57 ± 0.13
Donepezil	12.7 ± 0.9	nd	nd	nd
Tacrine	nd	2.2 ± 0.1 nM	nd	nd
Nimodipine	nd	nd	50± 10	nd
Melatonin	nd	nd	nd	2.45 ± 0.09

^a^ Every percentage value is the mean of a triple of at least two different experiments. ^b^ Data are expressed as Trolox equivalents and are the mean (*n* = 3) ± SEM. nd: not determined.

**Nrf2 transcriptional activation potencies of compounds BIGI 4b, BIGI 4d, BIGI 5b, and BIGI 5d**

The Nrf2-ARE-activating effect of **BIGI 4b**, **BIGI 4d**, **BIGI 5b**, and **BIGI 5d**, the most balanced compounds against cholinesterase inhibition, calcium channel blockade, and antioxidant power, was determined in vitro using a cell-based luciferase assay in the AREc32 cell line, which represents a good model for the redox-dependent activation of Nrf2 [31]. TBHQ was used as the positive control.

**Figure 2:**
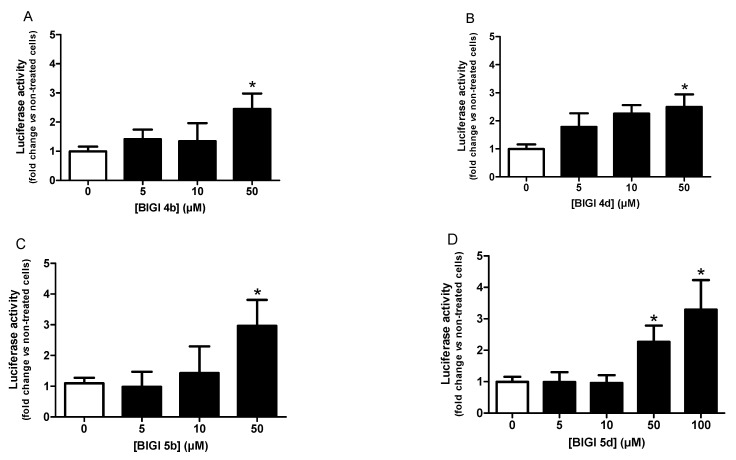

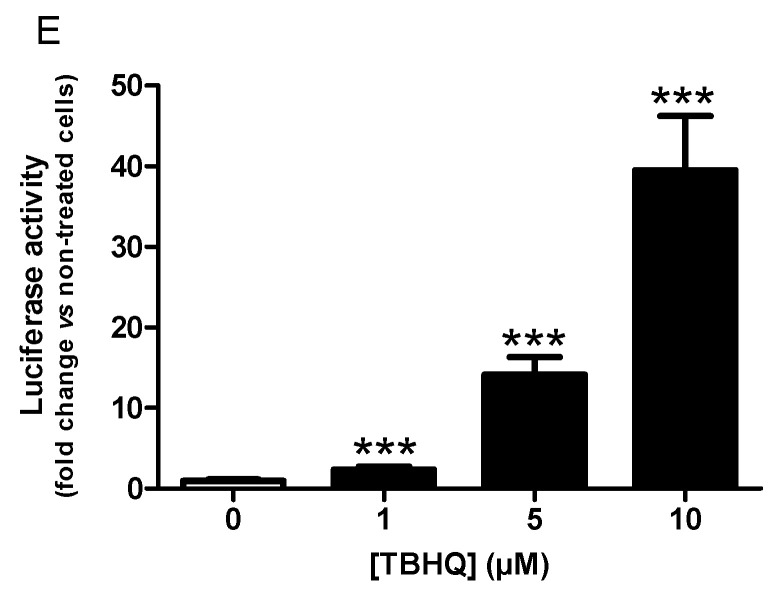
Nrf2 transcriptional activation potencies of compounds BIGI **4b** (**A**), BIGI **4d** (**B**), BIGI **5b** (**C**), and BIGI **5d** (**D**) or reference compound TBHQ I (**E**). Data are means ± SEM of at least 3 different experiments. * *p* ≤ 0.05 and *** *p* ≤ 0.001 with respect to non-treated cells (control cells).

AREc32 cells were treated over 24 h with increasing concentrations of each compound (5, 10, 50, 100 μM) and then luciferase activity was measured. Preliminarily, the cytotoxicity of compounds against AREc32 cells were evaluated, showing no toxicity until 50 μM for compounds **BIGI 4b**, **BIGI 4d**, and **BIGI 5b** and until 100 μM for compound **BIGI 5d**. As shown in Figure 2, compounds **BIGI 4b**, **BIGI 4d**, and **BIGI 5b** were able to successfully induce the Nrf2 transcriptional pathway at the concentrations of 5, 10, and 50 μM and up to 100 μM for compound **BIGI 5d**.

CD values (i.e., the concentration required to double the specific activity of the luciferase reporter) were calculated to compare the relative potencies. As shown in Table 2, compounds BIGI **4b**, BIGI **4d**, and BIGI **5b** were the most potent with a CD value equal to 7.1, 7.7, and 13.5 μM, respectively, compared with TBHQ which showed 0.8 μM. These compounds are thus only 9- to 17-fold less active than TBHQ, one of the most potent activators of Nrf2. Compound **5d** also showed an interesting CD value equal to 33.4 μM and was therefore 42 times less active than TBHQ but, for example, 2 times more active than melatonin (CD = 60 μM) [32] which is able to induce transcriptional pathway through several mechanisms [33]. 

#### 2.2.2. ADME Studies 

To predict the physicochemical properties of the compounds, “Data Warrior”, a physical and chemical property visualization and analysis software developed by Idorsia Pharmaceuticals Ltd., (Allschwil, Switzerland) was used. This software allows the prediction of drug-like properties using different parameters of Lipinski’s rule of five (molecular weight, LogP, LogS, H-Acceptors, H-Donors, and topological polar surface (TPSA)). As shown in Table 3, all the compounds presented MW values slightly higher than 500 g/mol, the value corresponding to the most orally administered drugs and used as a basis to establish Lipinski’s rule of five.

Lipophilicity is an important physiochemical property that determines whether the molecule will cross the biological membrane with predictive CLogP less than 5. Interestingly, all compounds showed suitable lipophilicity with CLogP values between 4.3929 and 4.9412, which compared very favorably to Donepezil which showed a CLogP equal to 4.2149. 

In order to gain more insight into the lipophilicity which plays a crucial role in determining the solubility of drug candidates in the biological system, we also calculated the log S values of these compounds. Table 3 shows that the CLogS of these compounds slightly exceed the limits set for this parameter and are comparable very favorably to Donepezil.

The number of donor and acceptor hydrogen bonds is in agreement with Lipinski’s rule of five. The number of donor hydrogen bonds is lower than 5 and the number of acceptors is also lower than 10 for all compounds. 

TPSA corresponds to the van der Waals surface of the molecule’s polar atoms (usually oxygen and nitrogen) and their attached hydrogens. A polar surface area no greater than 140 Å^2^ as suggested by Veber’s Rule. Interestingly, all the compounds have a TPSA of < 100 Å^2^. Moreover, compounds 4a-d exhibit lower PSA than compounds 5a-d.

Data Warrior also calculates “Druglikeness” as a qualitative concept to predict whether synthesized compounds are drug-like. This parameter is calculated using data including LogP, LogS, and molar mass, but also using other parameters such as the presence of structures with specific pharmacological properties (such as enones that can be mutagenic and carcinogenic). It can be noticed that all compounds have an interesting “Druglikeness” prediction even when they have a molecular weight higher than 500 g/mol. 

## 3. Materials and Methods

Melting points (°C) were determined with a Kofler hot bench and are uncorrected. Analytical thin-layer chromatography (TLC) on silica-gel-precoated aluminum sheets (Type 60 F254, 0.25 mm thickness; from Merck, Darmstadt, Germany) was employed to follow the progress of the reactions and to check the purity and homogeneity of the synthesized products. Nuclear magnetic resonance spectra (NMR) were recorded on a BRUCKER DRX-400 AVANCE spectrometer (at 400 MHz for ^1^H and 100 MHz for ^13^C), using dimethylsulfoxide (DMSO-d6) as the solvent and tetramethylsilane (TMS) as the internal standard. The chemical shifts are expressed in parts per million (ppm) and the multiplicities of ^1^H NMR signals were designated as follows: s: singlet; d: doublet; t: triplet; q: quartet; and m: multiplet, and coupling constants were expressed in hertz (Hz). High-resolution mass spectra (HRMS) were recorded using a Bruker micrOTOF-Q II spectrometer (Bruker Daltonics) in positive electrospray ionization time-of-flight mode at UCA Clermont Ferrand, France.

### 3.1. General Procedure for the Synthesis of Ureas and Thioureas 

Ureas and thioureas were prepared according to the literature [26]. To a solution of the appropriate amine (1 eq, 6.9 mmol) in dichloromethane (13 mL), a solution of benzoyl isocyanate or benzoyl isothiocyanate (1.2 eq) in dichloromethane (26 mL) was added dropwise at 0 °C. The mixture was stirred for 1h at room temperature. The solvent was then evaporated under reduced pressure and the residue triturated in Et_2_O and filtrated. It was dissolved in a mixture of iPrOH (26 mL) and aqueous NaOH (26 mL, 3.75M). The mixture was stirred for 72 h. The alcohol was evaporated and the aqueous phase extracted three times with AcOEt. The organic phases were collected, washed with brine, dried over anhydrous sodium sulfate, and evaporated. The residue was triturated in ether to give the desired compound. 

#### 3.1.1. 1-((1-Benzylpiperidin-4-yl)methyl)urea (2a)

Yield: 41% ^1^H NMR (400 MHz, CDCl_3_) δ 7.36–7.30 (m, 4H), 7.28–7.23 (m, 1H), 5.03 (bs, 1H), 4.60 (s, 2H), 3.50 (s, 2H), 3.05 (t, *J* = 6.2 Hz, 2H), 2.95–2.85 (m, 2H), 1.95 (td, *J* = 11.7, 2.1 Hz, 2H), 1.74–1.62 (m, 2H), 1.57–1.41 (m, 1H), 1.37–1.21 (m, 2H). ^13^C NMR (101 MHz, CDCl_3_) δ 159.10, 138.48, 129.33, 128.31, 127.12, 63.49, 53.51, 46.33, 36.49, 29.96.

#### 3.1.2. 1-(2-(1-Benzylpiperidin-4-yl)ethyl)urea (2b)

Yield: 43%. ^1^H NMR (400 MHz, CDCl_3_) δ 7.36–7.30 (m, 4H), 7.28–7.23 (m, 1H), 4.67 (bs, 1H), 4.46 (bs, 2H), 3.50 (s, 2H), 3.25–3.13 (m, 2H), 2.94–2.82 (m, 2H), 2.03–1.87 (m, 2H), 1.73–1.59 (m, 2H), 1.50–1.40 (m, 2H), 1.37–1.22 (m, 3H). ^13^C NMR (101 MHz, CDCl_3_) δ 158.75, 129.91, 128.60, 127.91, 62.97, 53.56, 38.33, 36.46, 33.09, 31.37.

#### 3.1.3. 1-((1-Benzylpiperidin-4-yl)methyl)thiourea (2c)

Yield: 35%. ^1^H NMR (400 MHz, MeOD) δ 7.35–7.30 (m, 4H), 7.30–7.23 (m, 1H), 3.52 (s, 2H), 3.43–3.35 (m, 1H), 3.05–2.96 (m, 1H), 2.95–2.87 (m, 2H), 2.08–1.95 (m, 2H), 1.79–1.65 (m, 2H), 1.65–1.54 (m, 1H), 1.39–1.20 (m, 2H). ^13^C NMR (101 MHz, MeOD) δ 138.32, 130.87, 129.28, 128.43, 64.28, 54.28, 51.29, 36.98, 30.46.

#### 3.1.4. 1-(2-(1-Benzylpiperidin-4-yl)ethyl)urea (2d)

Yield: 53%. ^1^H NMR (400 MHz, CDCl_3_) δ 7.34–7.27 (m, 4H), 7.26–7.21 (m, 1H), 6.28 (bs, 1H), 5.82 (bs, 2H), 3.48 (s, 2H), 3.16 (bs, 1H), 2.94–2.81 (m, 2H), 1.94 (t, *J* = 11.5 Hz, 2H), 1.87–1.74 (m, 1H), 1.70–1.59 (m, 2H), 1.58–1.48 (m, 2H), 1.43–1.19 (m, 3H). ^13^C NMR (101 MHz, CDCl_3_) δ 138.33, 129.22, 128.19, 127.00, 63.40, 53.57, 33.23, 32.07.

#### 3.1.5. Synthesis of 4-(prop-2-yn-1-yloxy)benzaldehyde (3a)

The aldehyde 3a was synthesized according to the literature [34]. A suspension of 4-hydroxybenzaldehyde (1 eq, 1.5 g) and potassium carbonate (1.3 eq, 2.2 g) in acetone (30 mL) was refluxed for 30 min. Once cooled to room temperature, 3-bromoprop-1-yne (1.6 eq, 1.5 mL) was added slowly and the resulting mixture was refluxed for 2h. The solvent was then evaporated and the residue solubilized in water and extracted three times with AcOEt. The organic phases were collected and dried over anhydrous sodium sulfate. Activated carbon was added and the solution left under stirring at 40 °C for 15 min. It was then filtrated on Celite. The filtrate was evaporated and the residue recrystallized using a mixture of AcOEt/hexane (1:2) to give the desired aldehyde.

Yield: 83%. ^1^H NMR (400 MHz, CDCl_3_) δ 9.90 (s, 1H), 7.85 (d, *J* = 8.7 Hz, 2H), 7.09 (d, *J* = 8.7 Hz, 2H), 4.78 (d, *J* = 2.4 Hz, 2H), 2.57 (t, *J* = 2.4 Hz, 1H). ^13^C NMR (101 MHz, CDCl_3_) δ 190.88, 162.49, 132.01, 130.73, 115.30, 77.67, 76.49, 56.07.

#### 3.1.6. Synthesis of 4-(but-3-yn-1-yloxy)benzaldehyde (3b)

The aldehyde 3b was synthesized according to the literature [25]. A solution of 4-hydroxybenzaldehyde (1 eq, 1 g), triphenylphosphine (2 eq, 4.3 g), and but-3-yn-1-ol (1.5 eq, 0.93 mL) in THF (60 mL) was cooled to 0°C. Diisopropyl azodicarboxylate (1.5 eq, 4.418 mL) was added dropwise and the mixture left under stirring at room temperature overnight. After evaporation of the solvent, the residue was solubilized in AcOEt, washed with NaOH (1M) and brine, dried over anhydrous Na_2_SO_4_, and evaporated. The residue was triturated with hexane and filtered. The filtrate was evaporated and purified by flash column chromatography using a mixture of hexane/AcOEt (90:10) to give the desired aldehyde.

Yield: 37%. ^1^H NMR (400 MHz, CDCl_3_) δ 9.92 (s, 1H), 7.86 (d, *J* = 8.8 Hz, 2H), 7.04 (d, *J* = 8.7 Hz, 2H), 4.20 (t, *J* = 6.9 Hz, 2H), 2.75 (td, *J* = 6.9, 2.7 Hz, 2H), 2.08 (t, *J* = 2.6 Hz, 1H). ^13^C NMR (101 MHz, CDCl_3_) δ 190.91, 163.55, 132.14, 130.38, 114.97, 79.98, 70.39, 66.34, 19.57.

### 3.2. Synthesis of the Biginelli Products 

#### 3.2.1. General Procedure for the Synthesis of Biginelli Products

The appropriate aldehyde (1 eq) and urea or thiourea were solubilized in glacial acetic acid and the resulting mixture was stirred at room temperature for 2 h. Ethyl acetoacetate (1.2 eq) and sodium hydrogen sulfate (1 eq) were added and the suspension left under stirring for 48h at room temperature, then refluxed for 4h. The catalyst was filtered. The filtrate was evaporated and purified by flash column chromatography using a mixture of CH_2_Cl_2_/MeOH/NH_3_ (95:4.05:0.05) to obtain the desired hybrid.

#### 3.2.2. Ethyl 1-((1-benzylpiperidin-4-yl)methyl)-6-methyl-2-oxo-4-(4-(prop-2-yn-1-yloxy)phenyl)-1,2,3,4-tetrahydropyrimidine-5-carboxylate (BIGI 4a) 

Yield: 28%. ^1^H NMR (400 MHz, CDCl_3_) δ 7.35–7.29 (m, 4H), 7.27–7.21 (m, 1H), 7.18 (d, *J* = 8.7 Hz, 2H), 6.85 (d, *J* = 8.7 Hz, 2H), 6.38 (d, *J* = 3.1 Hz, 1H), 5.38 (d, *J* = 3.5 Hz, 1H), 4.64 (d, *J* = 2.4 Hz, 2H), 4.15 (q, *J* = 7.1 Hz, 2H), 4.03 (dd, *J* = 14.3, 7.1 Hz, 1H), 3.47 (s, 2H), 3.31 (dd, *J* = 14.4, 6.6 Hz, 1H), 2.85–2.74 (m, 2H), 2.50 (s, 3H), 2.45 (t, *J* = 2.4 Hz, 1H), 1.89–1.78 (m, 2H), 1.54–1.35 (m, 3H), 1.29–1.15 (m, 5H). ^13^C NMR (101 MHz, CDCl_3_) δ 166.37, 157.02, 154.41, 149.18, 138.55, 136.38, 129.14, 128.26, 127.38, 127.02, 114.84, 105.62, 78.54, 75.72, 63.27, 60.36, 55.87, 53.33, 52.54, 47.26, 37.03, 29.97, 29.87, 16.48, 14.32. HRMS (ESI, M+H^+^) calcd for C_30_H_36_N_3_O_4_: 502.27003. Found: 502.26923.

#### 3.2.3. Ethyl 1-(2-(1-benzylpiperidin-4-yl)ethyl)-6-methyl-2-oxo-4-(4-(prop-2-yn-1-yloxy)phenyl)-1,2,3,4-tetrahydropyrimidine-5-carboxylate (BIGI 4b)

Yield: 13%. %. ^1^H NMR (400 MHz, CDCl_3_) δ 7.36–7.27 (m, 5H), 7.16 (d, *J* = 8.7 Hz, 2H), 6.88 (d, *J* = 8.7 Hz, 2H), 5.85 (d, *J* = 3.1 Hz, 1H), 5.30 (d, *J* = 3.1 Hz, 1H), 4.66 (d, *J* = 2.4 Hz, 2H), 4.09 (q, *J* = 7.1 Hz, 2H), 4.00–3.88 (m, 1H), 3.63 (s, 2H), 3.62–3.53 (m, 1H), 3.04–2.93 (m, 2H), 2.52 (t, *J* = 2.4 Hz, 1H), 2.49 (s, 3H), 2.09–2.04 (m, 1H), 2.02–1.96 (m, 1H), 1.77–1.68 (m, 1H), 1.66–1.57 (m, 1H), 1.57–1.47 (m, 1H), 1.47–1.32 (m, 3H), 1.30–1.20 (m, 1H), 1.17 (t, *J* = 7.1 Hz, 3H). ^13^C NMR (101 MHz, CDCl_3_) δ 166.22, 157.23, 153.83, 148.38, 136.75, 130.05, 128.54, 127.87, 127.61, 115.06, 105.27, 78.56, 75.83, 62.47, 60.37, 55.97, 53.31, 52.99, 52.94, 40.38, 36.20, 33.03, 31.30, 30.93, 16.15, 14.29. HRMS (ESI, M+H^+^) calcd for C_31_H_38_N_3_O_4_: 516.28568. Found: 516.28643. 

#### 3.2.4. Ethyl 1-((1-benzylpiperidin-4-yl)methyl)-4-(4-(but-3-yn-1-yloxy)phenyl)-6-methyl-2-oxo-1,2,3,4-tetrahydropyrimidine-5-carboxylate (BIGI 4c)

Yield: 39%. ^1^H NMR (400 MHz, CDCl_3_) δ 7.36–7.27 (m, 5H), 7.15 (d, *J* = 8.6 Hz, 2H), 6.80 (d, *J* = 8.7 Hz, 2H), 5.77 (d, *J* = 3.1 Hz, 1H), 5.35 (d, *J* = 3.3 Hz, 1H), 4.12 (q, *J* = 7.1 Hz, 2H), 4.04 (t, *J* = 7.0 Hz, 2H), 3.99 (dd, *J* = 14.4, 6.7 Hz, 1H), 3.56 (s, 2H), 3.32 (dd, *J* = 14.4, 6.8 Hz, 1H), 2.92–2.81 (m, 2H), 2.65 (td, *J* = 7.0, 2.7 Hz, 2H), 2.47 (s, 3H), 2.04 (t, *J* = 2.6 Hz, 1H), 2.01–1.87 (m, 2H), 1.59–1.38 (m, 3H), 1.37–1.23 (m, 2H), 1.19 (t, *J* = 7.1 Hz, 3H). ^13^C NMR (101 MHz, CDCl_3_) δ 166.35, 158.08, 154.19, 148.83, 135.89, 129.64, 128.46, 127.48, 114.74, 105.77, 80.45, 70.13, 66.17, 62.81, 60.47, 52.94, 52.91, 47.21, 36.66, 29.38, 19.66, 16.61, 14.34, 14.27. HRMS (ESI, M+H^+^) calcd for C_31_H_38_N_3_O_4_: 516.28568. Found: 516.28524. 

#### 3.2.5. Ethyl 1-(2-(1-benzylpiperidin-4-yl)ethyl)-4-(4-(but-3-yn-1-yloxy)phenyl)-6-methyl-2-oxo-1,2,3,4-tetrahydropyrimidine-5-carboxylate (BIGI 4d)

Yield: 34%. ^1^H NMR (400 MHz, CDCl_3_) δ 7.43–7.28 (m, 5H), 7.14 (d, *J* = 8.7 Hz, 2H), 6.81 (d, *J* = 8.7 Hz, 2H), 5.76 (bs, 1H), 5.30 (s, 1H), 4.14–4.02 (m, 4H), 4.01–3.87 (m, 1H), 3.72 (s, 2H), 3.63–3.52 (m, 1H), 3.13–2.98 (m, 2H), 2.66 (td, *J* = 7.0, 2.7 Hz, 2H), 2.49 (s, 3H), 2.12 (t, *J* = 9.8 Hz, 2H), 2.03–2.02 (m, 1H), 1.82–1.72 (m, 1H), 1.69–1.61 (m, 1H), 1.60–1.39 (m, 4H), 1.27–1.20 (m, 1H), 1.17 (t, *J* = 7.1 Hz, 3H). ^13^C NMR (101 MHz, CDCl_3_) δ 166.21, 158.14, 153.83, 148.22, 136.25, 130.34, 128.72, 127.65, 114.85, 105.39, 80.46, 70.12, 66.21, 62.19, 60.40, 53.36, 52.80, 40.28, 36.07, 32.80, 30.86, 30.44, 19.67, 16.16, 14.30. HRMS (ESI, M+H^+^) calcd for C_32_H_40_N_3_O_4_: 530.30133. Found: 530.30129. 

#### 3.2.6. Ethyl 1-((1-benzylpiperidin-4-yl)methyl)-6-methyl-4-(4-(prop-2-yn-1-yloxy)phenyl)-2-thioxo-1,2,3,4-tetrahydropyrimidine-5-carboxylate (BIGI 5a)

Yield: 10%. ^1^H NMR (400 MHz, CDCl_3_) δ 7.94 (s, 1H), 7.43–7.37 (m, 5H), 7.16 (d, *J* = 8.6 Hz, 2H), 6.85 (d, *J* = 8.8 Hz, 2H), 5.45 (d, *J* = 4.8 Hz, 1H), 4.92–4.76 (m, 1H), 4.65 (d, *J* = 2.3 Hz, 2H), 4.33–4.15 (m, 2H), 3.87 (s, 2H), 3.56–3.38 (m, 1H), 3.21–3.09 (m, 1H), 2.99–2.86 (m, 1H), 2.59 (t, *J* = 2.3 Hz, 1H), 2.47 (s, 3H), 2.42–2.32 (m, 1H), 2.15–2.04 (m, 1H), 1.75–1.61 (m, 1H), 1.60–1.49 (m, 2H), 1.46–1.36 (m, 1H), 1.29 (t, *J* = 7.1 Hz, 3H), 1.23–1.16 (m, 1H). ^13^C NMR (101 MHz, CDCl_3_) δ 179.71, 165.85, 157.13, 147.08, 134.46, 130.96, 129.76, 129.30, 127.14, 114.85, 110.48, 78.46, 76.35, 61.25, 55.94, 52.31, 51.87, 51.78, 51.31, 34.72, 26.54, 17.00, 14.38. HRMS (ESI, M+H^+^) calcd for C_31_H_38_N_3_O_4_: 516.28568. Found: 516.28643. 

#### 3.2.7. Ethyl 1-(2-(1-benzylpiperidin-4-yl)ethyl)-6-methyl-4-(4-(prop-2-yn-1-yloxy)phenyl)-2-thioxo-1,2,3,4-tetrahydropyrimidine-5-carboxylate (BIGI 5b)

Yield: 20%. ^1^H NMR (400 MHz, CDCl_3_) δ 7.53 (bs, 1H), 7.48–7.39 (m, 5H), 7.16 (d, *J* = 8.7 Hz, 2H), 6.90 (d, *J* = 8.7 Hz, 2H), 5.33 (d, *J* = 4.1 Hz, 1H), 4.97–4.77 (m, 1H), 4.68 (t, *J* = 2.2 Hz, 2H), 4.17 (q, *J* = 7.1 Hz, 2H), 3.98–3.84 (m, 3H), 3.27–3.09 (m, 2H), 2.55 (t, *J* = 2.4 Hz, 1H), 2.53 (s, 3H), 2.40–2.21 (m, 2H), 2.04–1.95 (m, 1H), 1.73–1.50 (m, 4H), 1.49–1.39 (m, 1H), 1.22 (t, *J* = 7.1 Hz, 3H), 1.11–0.97 (m, 1H). ^13^C NMR (101 MHz, CDCl_3_) δ 179.13, 165.73, 157.46, 145.83, 135.00, 130.99, 129.79, 129.37, 127.64, 115.17, 109.61, 78.44, 76.17, 61.43, 61.05, 56.12, 52.76, 52.38, 45.39, 35.35, 31.20, 29.49, 28.93, 16.50, 14.30. HRMS (ESI, M+H^+^) calcd for C_31_H_38_N_3_O_3_S: 532.26284. Found: 532.26302. 

#### 3.2.8. Ethyl 1-((1-benzylpiperidin-4-yl)methyl)-4-(4-(but-3-yn-1-yloxy)phenyl)-6-methyl-2-thioxo-1,2,3,4-tetrahydropyrimidine-5-carboxylate (BIGI 5c) 

Yield: 15%. 1H NMR (400 MHz, CDCl3) δ 8.07 (bs, 1H), 7.42–7.28 (m, 5H), 7.13 (d, *J* = 8.6 Hz, 2H), 6.77 (d, *J* = 8.7 Hz, 2H), 5.41 (d, *J* = 4.5 Hz, 1H), 5.02–4.78 (m, 1H), 4.30–4.12 (m, 2H), 4.01 (t, *J* = 6.9 Hz, 2H), 3.69 (s, 2H), 3.55–3.37 (m, 1H), 3.05–2.92 (m, 1H), 2.89–2.76 (m, 1H), 2.65 (td, *J* = 6.9, 2.6 Hz, 2H), 2.45 (s, 3H), 2.16–2.07 (m, 1H), 2.04 (t, *J* = 2.6 Hz, 1H), 2.00–1.82 (m, 1H), 1.65–1.53 (m, 1H), 1.50–1.31 (m, 3H), 1.27–1.22 (m, 4H), 1.12–0.92 (m, 1H). 13C NMR (101 MHz, CDCl3) δ 179.44, 165.87, 158.11, 146.80, 134.00, 130.25, 128.76, 127.24, 114.64, 109.84, 80.37, 70.23, 66.16, 62.12, 61.04, 52.38, 52.11, 51.86, 35.76, 19.64, 16.84, 14.33. HRMS (ESI, M+H^+^) calcd for C_31_H_38_N_3_O_3_S: 532.26284. Found: 532.26335. 

#### 3.2.9. Ethyl 1-(2-(1-benzylpiperidin-4-yl)ethyl)-4-(4-(but-3-yn-1-yloxy)phenyl)-6-methyl-2-thioxo-1,2,3,4-tetrahydropyrimidine-5-carboxylate (BIGI 5d)

Yield: 14%. ^1^H NMR (400 MHz, CDCl_3_) δ 7.70–7.54 (m, 1H), 7.54–7.47 (m, 2H), 7.46–7.40 (m, 3H), 7.14 (d, *J* = 8.6 Hz, 2H), 6.83 (d, *J* = 8.7 Hz, 2H), 5.32 (d, *J* = 3.9 Hz, 1H), 4.89–4.76 (m, 1H), 4.16 (q, *J* = 7.1 Hz, 2H), 4.10–3.96 (m, 4H), 3.95–3.84 (m, 1H), 3.35–3.22 (m, 2H), 2.66 (td, *J* = 6.8, 2.7 Hz, 2H), 2.52 (s, 3H), 2.48–2.28 (m, 2H), 2.03 (t, *J* = 2.6 Hz, 1H), 1.90–1.54 (m, 4H), 1.53–1.41 (m, 1H), 1.21 (t, *J* = 7.1 Hz, 3H), 1.15–1.00 (m, 1H). ^13^C NMR (101 MHz, CDCl_3_) δ 178.90, 165.73, 158.35, 145.59, 134.57, 131.01, 129.89, 129.40, 127.70, 114.99, 109.44, 80.48, 70.23, 66.36, 61.29, 61.00, 52.91, 52.26, 45.43, 35.27, 31.30, 29.33, 28.78, 19.69, 16.51, 14.28. HRMS (ESI, M+H^+^) calcd for C_32_H_40_N_3_O_3_S: 546.27849. Found: 546.27740. 

### 3.3. Biological Evaluation

#### 3.3.1. hAChE and eqBuChE

The inhibitory capacity of the compounds on AChE biological activity was evaluated using the spectrometric method of Ellman [27]. Acetyl- or butyrylthiocholine iodide and 5,5-dithiobis- (2-nitrobenzoic) acid (DTNB) were purchased from Sigma Aldrich. Lyophilized BuChE from equine serum (Sigma Aldrich) was dissolved in 0.2 M phosphate buffer pH 7.4 to obtain enzyme solution stock with 2.5 units/mL enzyme activity. AChE from human erythrocytes (buffered aqueous solution, ≥500 units/mg protein (BCA), Sigma Aldrich) was diluted in 20 mM HEPES buffer, pH 8, 0.1% Triton X-100, to obtain an enzyme solution with 0.25 unit/mL enzyme activity. In the procedure, 100 µL of 0.3 mM DTNB dissolved in phosphate buffer pH 7.4 was added to the 96-well plate followed by 50 µL of test compound solution and 50 µL of enzyme (0.05 U final). After 5 min of preincubation at 25 °C, the reaction was then initiated by the injection of 50 µL of 10 mM acetyl- or butyrylthiocholine iodide solution. The hydrolysis of acetyl- or butyrylthiocholine was monitored by the formation of yellow 5-thio-2-nitrobenzoate anion as the result of the reaction of DTNB with thiocholine, released by the enzymatic hydrolysis of acetyl- or butyrylthiocholine, at a wavelength of 412 nm using a 96-well microplate reader (TECAN Infinite M200, Lyon, France). Test compounds were dissolved in analytical-grade DMSO. Donepezil was used as a reference standard. The rate of absorbance increase at 412 nm was followed every minute for 10 min. Assays were performed in singlicate during three independent tests, with a blank containing all components except acetyl- or butyrylthiocholine in order to account for non-enzymatic reaction. The reaction slopes were compared, and the percent inhibition due to the presence of test compounds was calculated by the following: 100 − (vi/v0 × 100) where vi is the rate calculated in the presence of inhibitor and v0 is the enzyme activity. The first screening of AChE and BuChE activity was carried out at a 10^−6^ or 10^−5^ M concentration of the compounds under study.

For the compounds with significant inhibition (≥50%), IC_50_ values were determined graphically by plotting the % inhibition versus the logarithm of six inhibitor concentrations in the assay solution using the GraphPad Prism 6.

#### 3.3.2. Calcium Channel Blockade

The evaluation of the calcium channel blockade of compounds BIGI **4a-d** and BIGI **5a-d** was carried out using FLIPR Calcium 6 indicator according to previously described protocols [35,36]. In brief, FLIPR-loaded SH-SY5Y cells were exposed to nimodipine (10 µM, reference inhibitor), DMSO (0.1%, vehicle), or our compounds of interest (10 µM) for 10 min. Calcium flux was triggered using KCl and CaCl_2_ (90 and 5 mM, respectively) and the resulting change in fluorescence was recorded (λ_Ex_ = 485 nm; λ_Em_ = 525 nm). Data were gathered in three independent experiments with eight technical replicates per experiment. Outlier detection by Grubbs’ test was performed and outlying values were excluded from further analysis.

#### 3.3.3. Oxygen Radical Absorbance Capacity Assay

The antioxidant activity of hybrids **BIGI 4a-d** and **BIGI 5a-d** was measured by ORAC-FL assay using fluorescein as a fluorescent probe. Briefly, fluorescein and antioxidant were incubated in a black 96-well microplate (Nunc) for 15 min at 37 °C. 2,2′-Azobis(amidinopropane) dihydrochloride was then added quickly using the built-in injector of a Varioskan Flash plate reader (Thermo Scientific, Waltham, MA, USA). The fluorescence was measured at 485 nm (excitation wavelength) and 535 nm (emission wavelength) each min for 1h. All the reactions were made in triplicate and at least three different assays were performed for each sample.

#### 3.3.4. Nrf2 Transcriptional Activation Potencies of Compounds **BIGI 4b**, **BIGI 4d**, **BIGI 5b**, and **BIGI 5d**

Treatment of stable ARE-luciferase reporter cells with the tested compounds and evaluation of the luciferase activity: The NRF2/ARE-luciferase reporter HEK293 stable cell line (Signosis, Santa Clara, CA, USA) was maintained in Dulbecco’s MEM high-glucose (DMEM) medium supplemented with 10% FBS and penicillin–streptomycin at 37 °C in 95% air/5% CO_2_. For treatment, the cells were seeded at a density of 2 × 10^4^ per well in 96-well white microtiter plates. After 48 h, the culture medium was replaced with fresh DMEM supplemented with 0.1% FBS containing different concentrations of the tested compounds or DMSO (0.1%) in duplicate. Luciferase activity was measured after 24 h of treatment using the Bright-Glo luciferase assay system (Promega) according to the manufacturer’s instructions.

**Treatment of stable ARE-luciferase reporter cells with the tested compounds and evaluation of the cellular viability:** The NRF2/ARE-luciferase reporter HEK293 stable cell line was seeded and treated as described for the luciferase activity determination, except that transparent culture plates were used instead of white plates. After 24 h of incubation with the tested compounds, the percent of cell viability was measured using MTT assay.

## 4. Conclusions

In the present study, we designed and synthesized via the Biginelli multicomponent reaction eight new compounds. From all the biological and physicochemical results gathered in this study, we identified compounds **BIGI 4b**, **BIGI 4d**, and **BIGI 5b** as new multitarget-directed ligands able to simultaneously address cholinesterase inhibition, calcium channel blockade, antioxidant power measured by ORAC assay, and the Nrf2-ARE-activating effect. In addition, these compounds showed suitable physicochemical properties and “Druglikeness” scores for druggability predicted by the Data Warrior software.

This study revealed that compounds **BIGI 4b**, **BIGI 4d**, and **BIGI 5b** may be promising agents for further research into the treatment of Alzheimer’s disease.

Work is now in progress in our laboratories to develop analogues with the best pharmacological profile. The results will be reported in due course.

## Figures and Tables

**Table 2:** Nrf2 induction potencies of compounds **BIGI 4b**, **BIGI 4d**, **BIGI 5b**, and **BIGI 5d** and reference compound TBHQ in Nrf2/ARE-luciferase reporter cells.

Compound	CD (µM)
**BIGI 4b**	7.1 ± 0.8
**BIGI 4d**	7.7 ± 2.9
**BIGI 5b**	13.5 ± 9.1
**BIGI 5d**	33.4 ± 4.8
TBHQ	0.8 ± 0.2

Data were plotted as concentration–response curves and fitted to a nonlinear equation. Data are expressed as the concentration required to double the specific luciferase reporter activity (CD) and are means ± SEM of at least 3 different experiments.

**Table 3:** Physicochemical properties of the synthesized compounds calculated by Data Warrior.

Name	Molweight (g/mol)	CLogP	CLogS	H-Donors	H-Acceptors	Druglikeness	TPSA (Å²)
**BIGI** **4a**	501.625	4.0324	−4.489	1	7	3.8852	71.11
**BIGI** **4b**	515.652	4.4868	−4.759	1	7	3.5578	71.11
**BIGI** **4c**	515.652	4.4868	−4.759	1	7	2.3145	71.11
**BIGI** **4d**	529.679	4.9412	−5.029	1	7	1.9887	71.11
**BIGI** **5a**	517.692	4.3929	−4.573	1	6	2.6429	86.13
**BIGI** **5b**	531.719	4.8473	−4.843	1	6	2.2966	86.13
**BIGI** **5c**	531.719	4.8473	−4.843	1	6	1.0706	86.13
**BIGI** **5d**	531.719	4.8473	−4.843	1	6	1.0706	86.13
Donepezil	379.498	4.2149	−4.347	0	4	5.6409	38.77
Selegiline	173.258	1.8326	−2.606	1	1	4.7279	12.03

## Data Availability

Not applicable.

## Supplementary Materials

The NMR spectras are available in the following supporting information which can be downloaded at: https://www.mdpi.com/article/10.3390/molecules28010071/s1. Figure S1: ^1^H NMR spectrum of compound 4a; Figure S2: ^13^C NMR spectrum of compound 4a; Figure S3: ^1^H NMR spectrum of compound 5a; Figure S4: ^13^C NMR spectrum of compound 5a; Figure S5: ^1^H NMR spectrum of compound 4b; Figure S6: ^13^C NMR spectrum of compound 4b; Figure S7: ^1^H NMR spectrum of compound 5b; Figure S8: ^13^C NMR spectrum of compound 5b; Figure S9: ^1^H NMR spectrum of compound 4c; Figure S10: ^13^C NMR spectrum of compound 4c; Figure S11: ^1^H NMR spectrum of compound 5c; Figure S12: ^13^C NMR spectrum of compound 5c; Figure S13: ^1^H NMR spectrum of compound 4d; Figure S14: ^13^C NMR spectrum of compound 4d; Figure S15: ^1^H NMR spectrum of compound 5d; Figure S16: ^13^C NMR spectrum of compound 5d.

## Sample Availability

Samples of the compounds are available from the authors.
