# Supplementary material for: Biginelli Reaction Synthesis of Novel Multitarget-Directed Ligands with Ca2+ Channel Blocking Ability, Cholinesterase Inhibition, Antioxidant Capacity, and Nrf2 Activation
Source: Molecules. 2022 Dec 22;28(1):71. doi: 10.3390/molecules28010071 (PMC9822022; doi:10.3390/molecules28010071)

SUPPORTING INFORMATION

Biginelli Reaction Synthesis of Novel Multitarget-Directed Ligands with Ca^{2+} Channel Blocking Ability, Cholinesterase Inhibition, Antioxidant Capacity, and Nrf2 Activation

Rim Malek [1,2], Alexey Simakov [3], Audrey Davis [4], Maciej Maj [5], Paul J. Bernard [1], Artur Wnorowski [5],
Helene Martin [3], José Marco-Contelles [6], Fakher Chabchoub [2], Patrick Dallemagne [4], Christophe Rochais [4], Krzysztof Jozwiak [5] and Lhassane Ismaili [1,*]

[1] Laboratoire LINC UR 481, Pôle de Chimie Médicinale, Univ. Franche-Comté, UFR Santé, 19, rue Ambroise Paré, F-25000 Besançon, France
[2] Laboratory of Applied Chemistry: Heterocycles, Lipids and Polymers, Faculty of Sciences of Sfax
University of Sfax, B. P 802, Sfax 3000, Tunisia; fakher.chabchoub@fss.usf.tn
[3] PEPITE EA4267, Univ. Franche-Comté, F-25000 Besançon, France
[4] Centre d'Etudes et de Recherche sur le Médicament de Normandie, Normandie Univ, Unicaen, CERMN, 14000 Caen, France
[5] Department of Biopharmacy, Medical University of Lublin, ul. W. Chodzki 4a, 20-093 Lublin, Poland
[6] Laboratory of Medicinal Chemistry (IQOG, CSIC) C/ Juan de la Cierva 3, 28006 Madrid, Spain
* Correspondence: lhassane.ismaili@univ-fcomte.fr

Table of Contents

Spectroscopic data of compounds 4a-d and 5a-d.

Figure S1: ^{1}H NMR spectrum of compound 4a

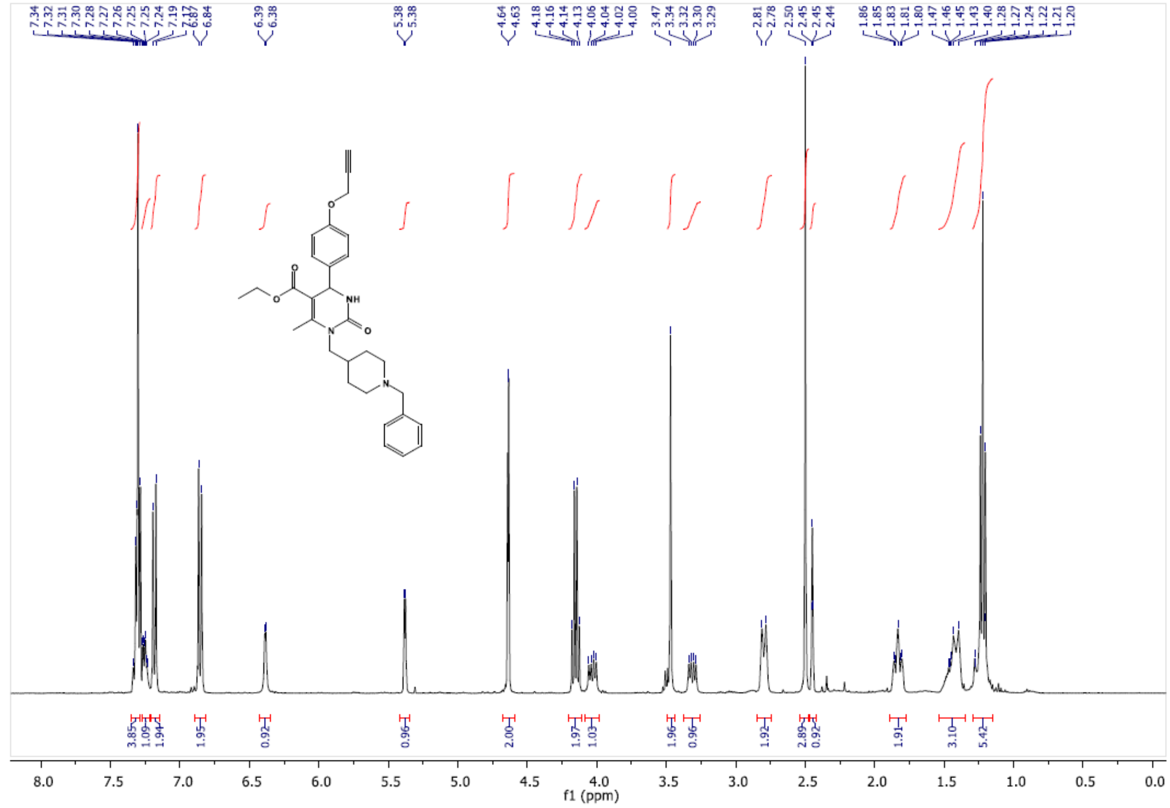

Figure S2: ^{13}C NMR spectrum of compound 4a

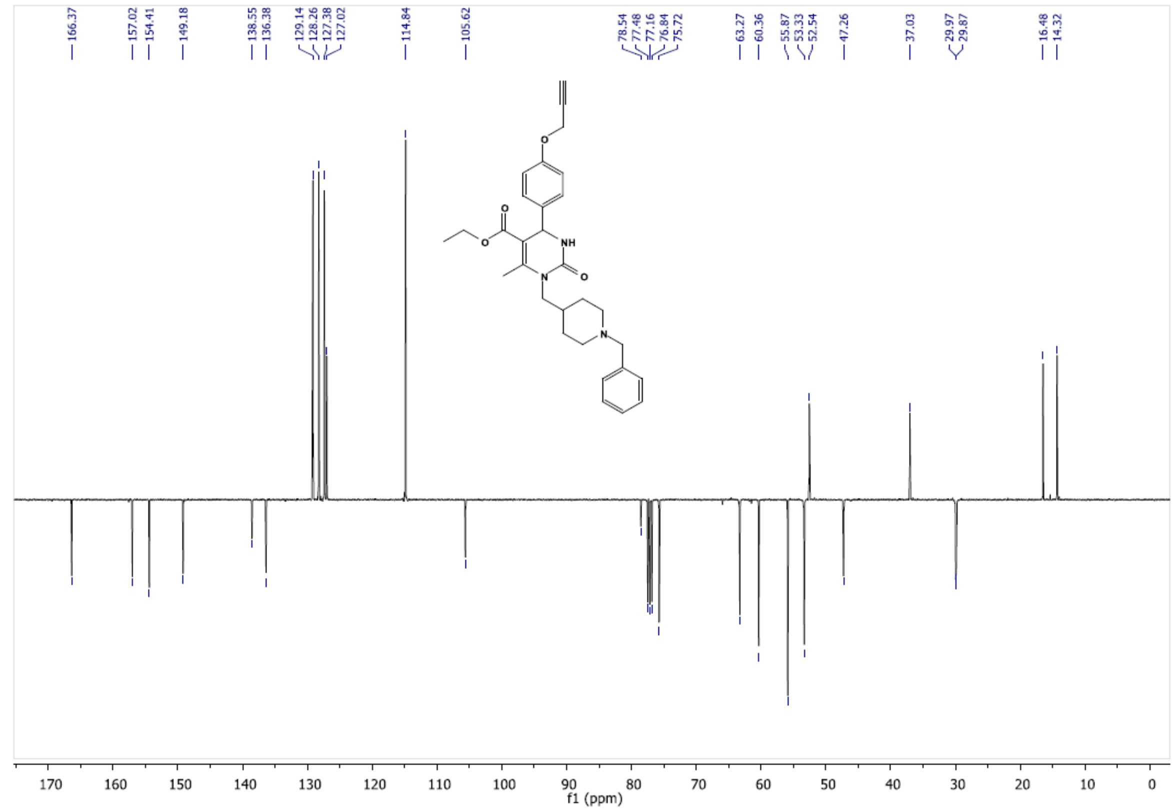

Figure S3: **^{1}H NMR spectrum of compound 5a**

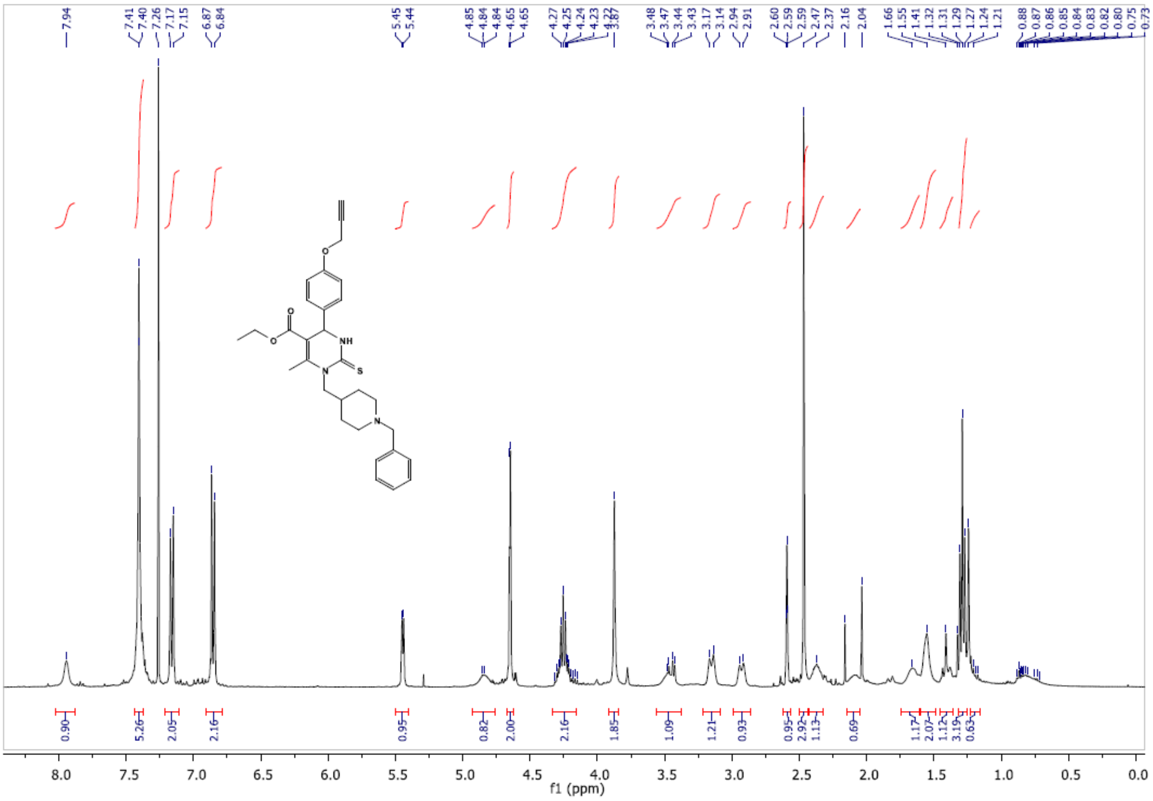

Figure S4: **^{13}C NMR spectrum of compound 5a**

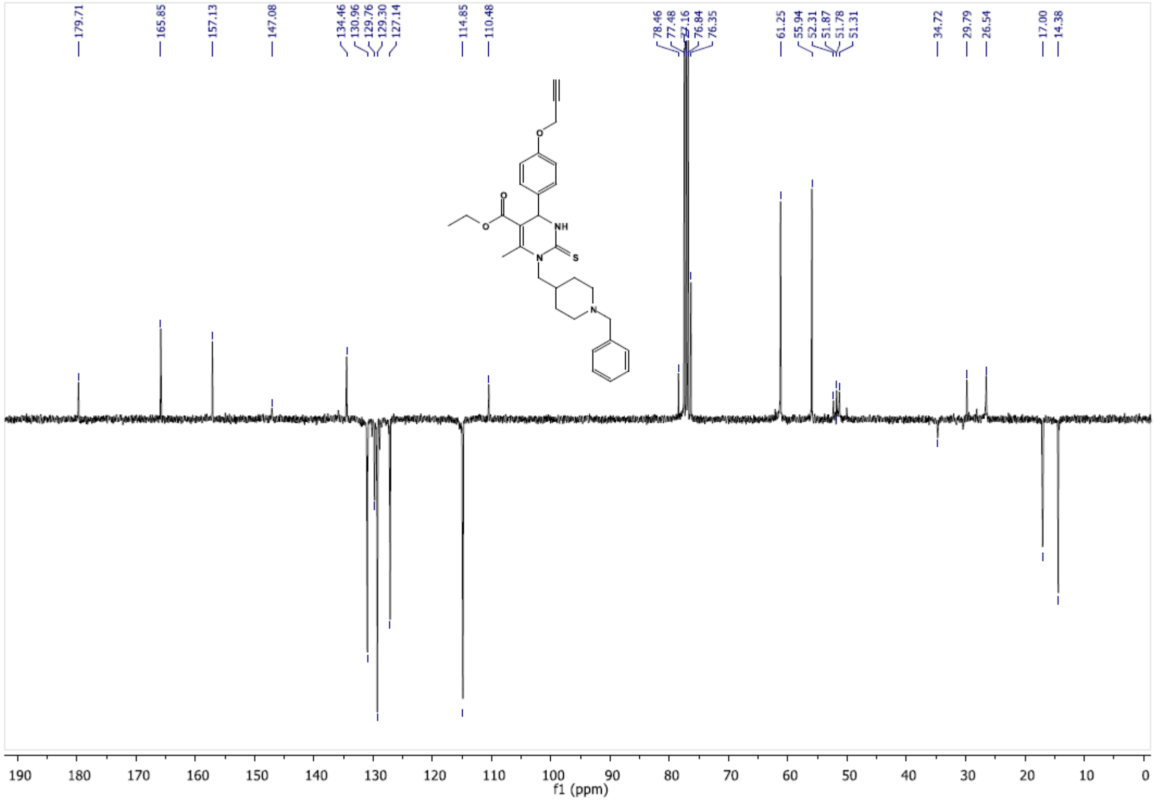

Figure S5: ^{1}H NMR spectrum of compound 4b

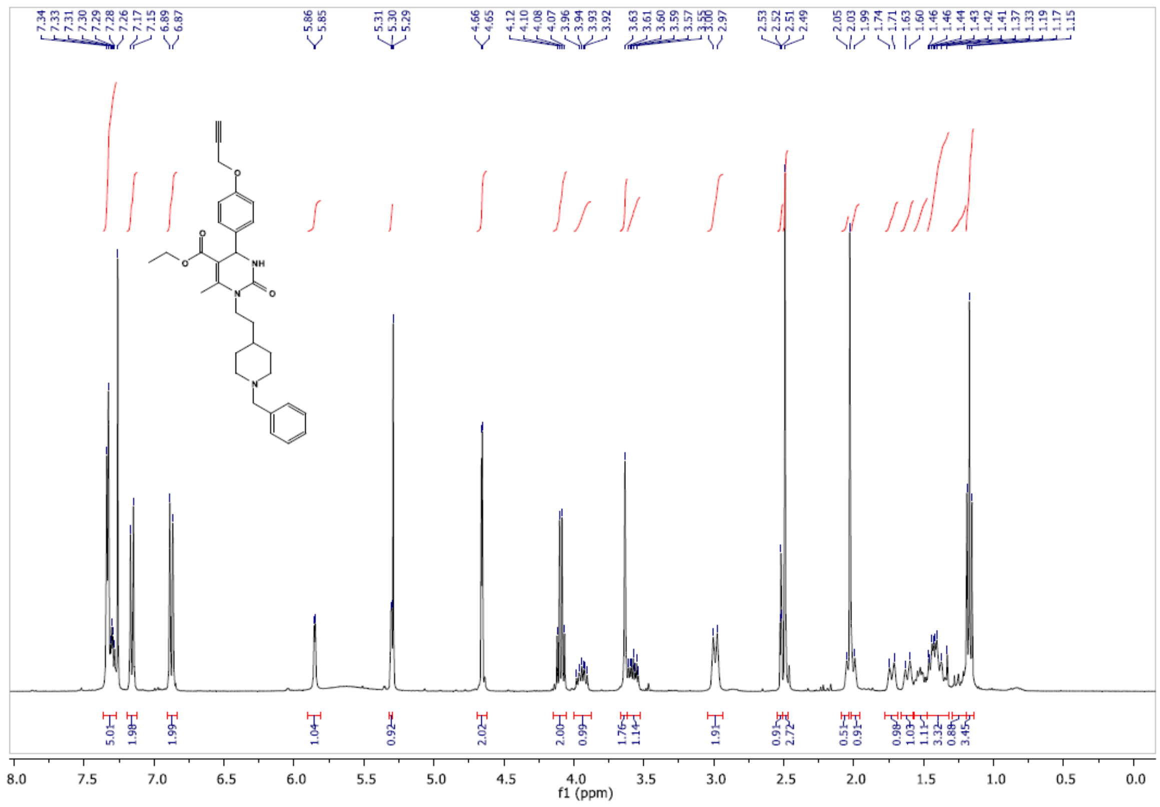

Figure S6: ^{13}C NMR spectrum of compound 4b

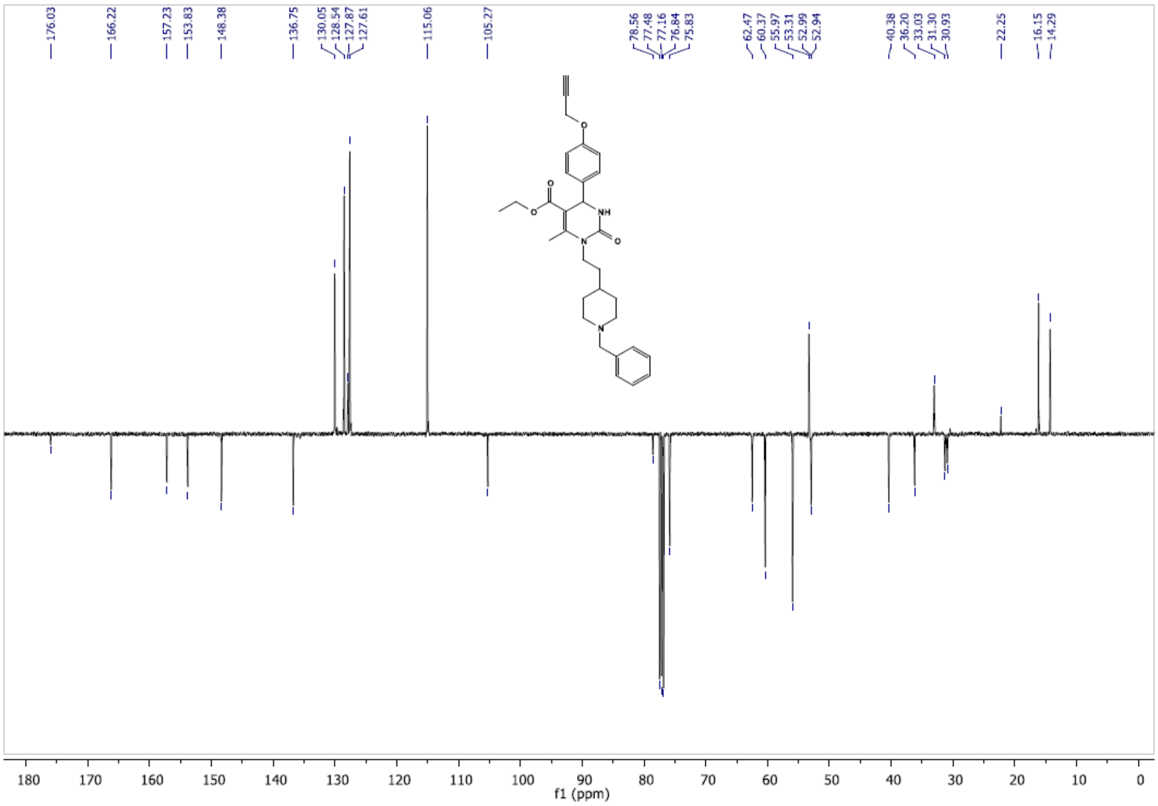

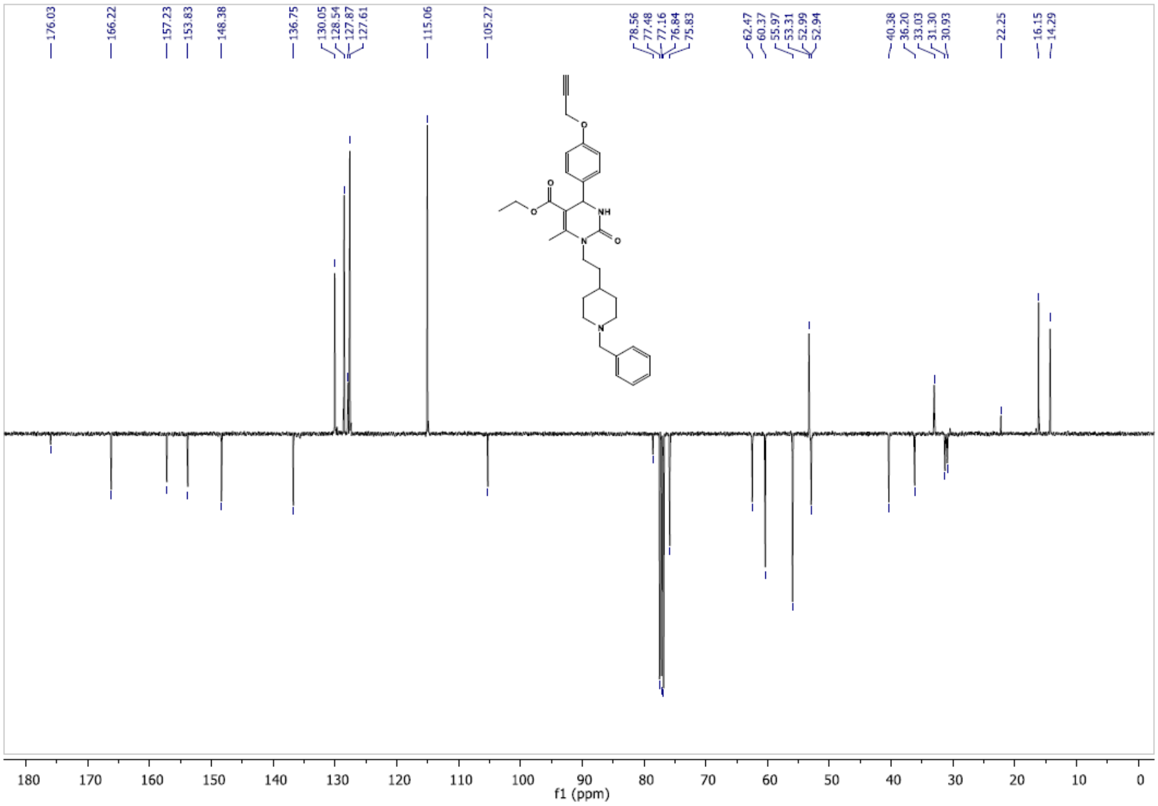

Figure S7: ¹H NMR spectrum of compound 5b

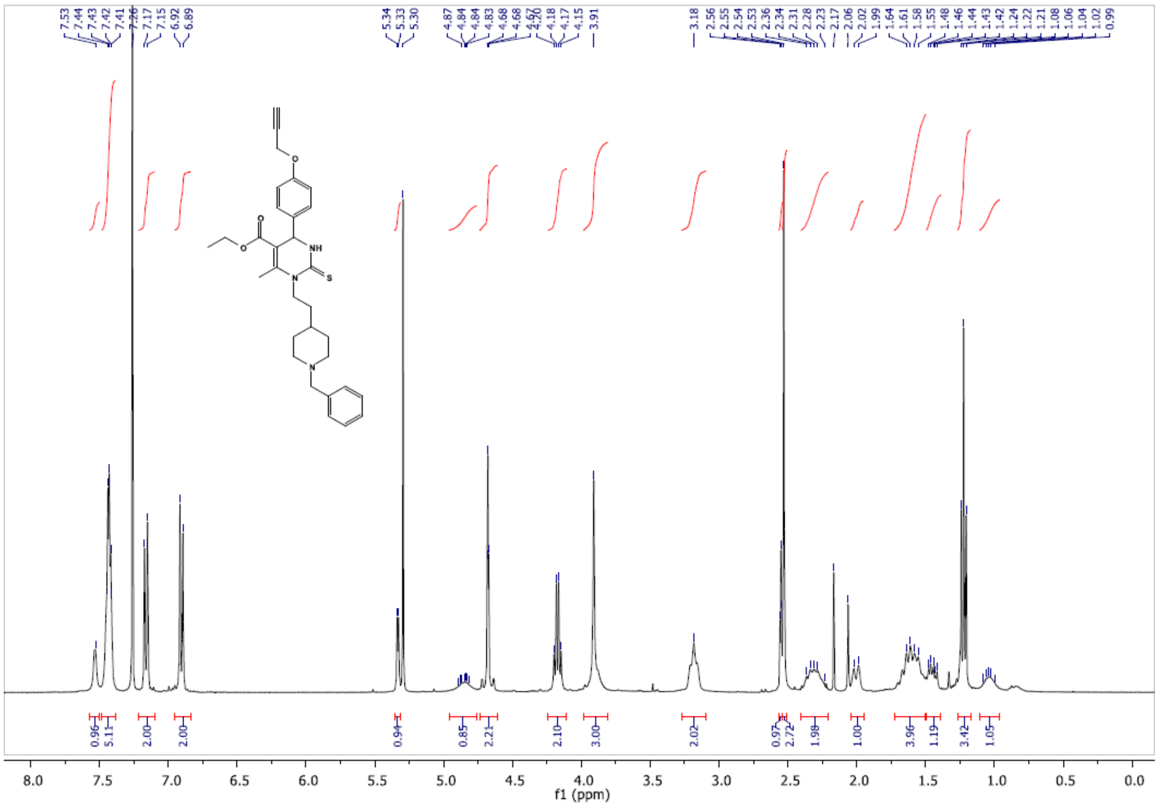

Figure S8: ¹³C NMR spectrum of compound 5b

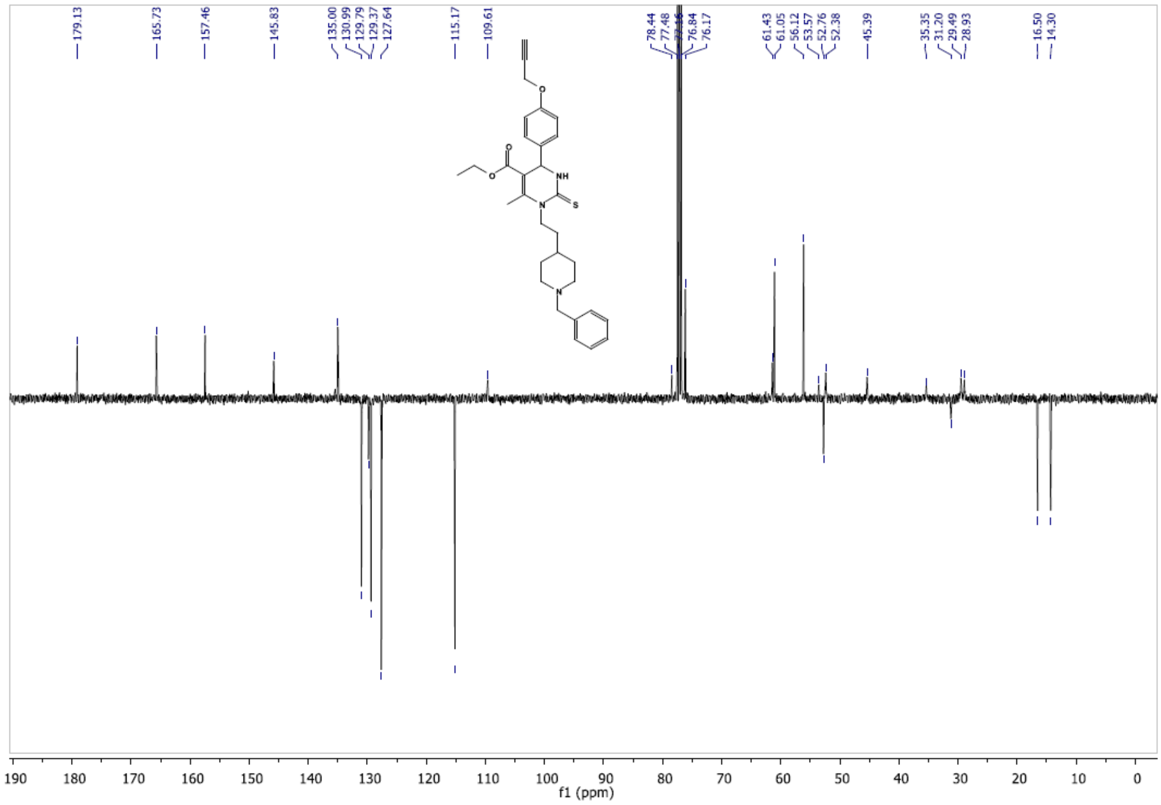

Figure S9: ^{1}H NMR spectrum of compound 4c

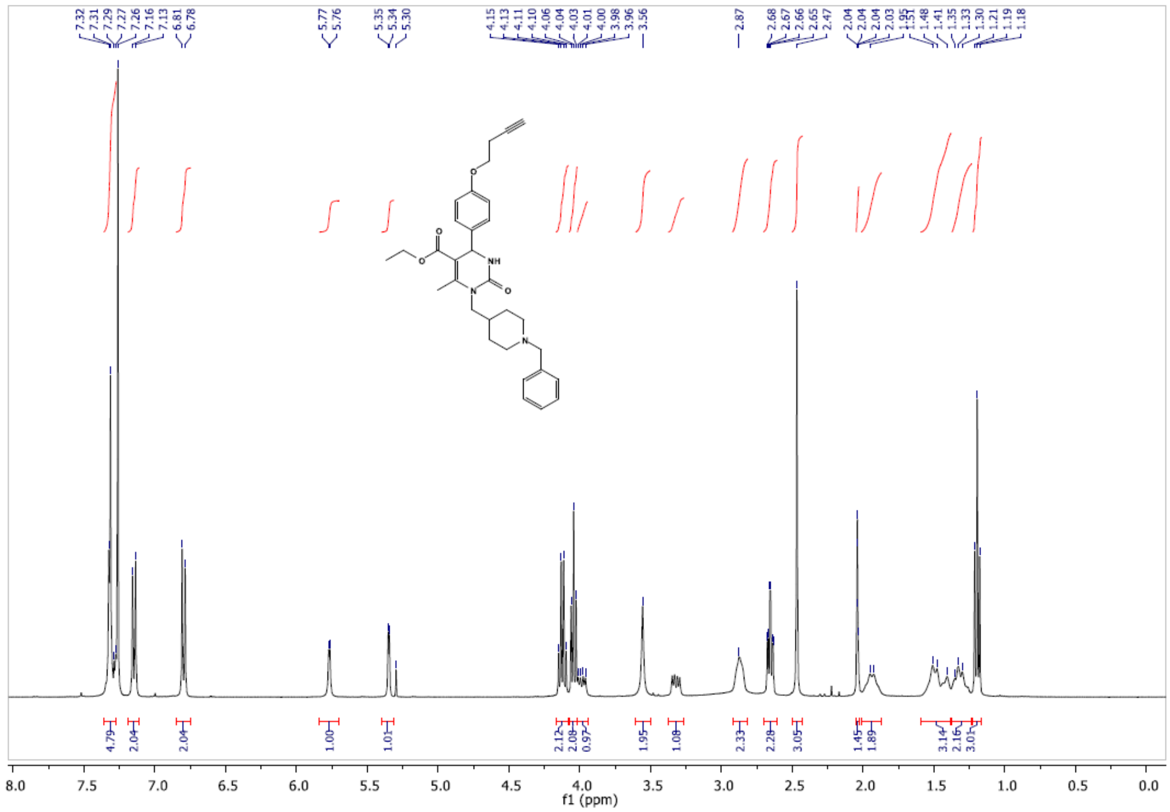

Figure S10: ^{13}C NMR spectrum of compound 4c

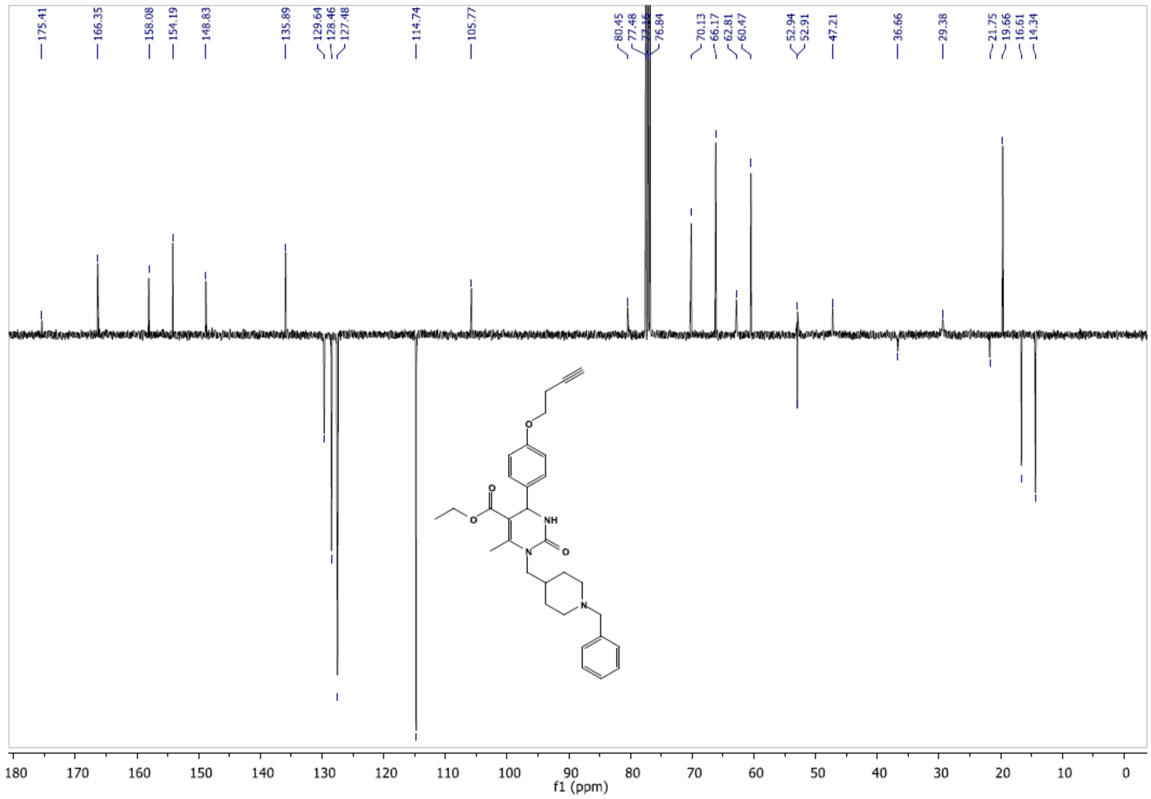

Figure S11: ^{1}H NMR spectrum of compound 5c

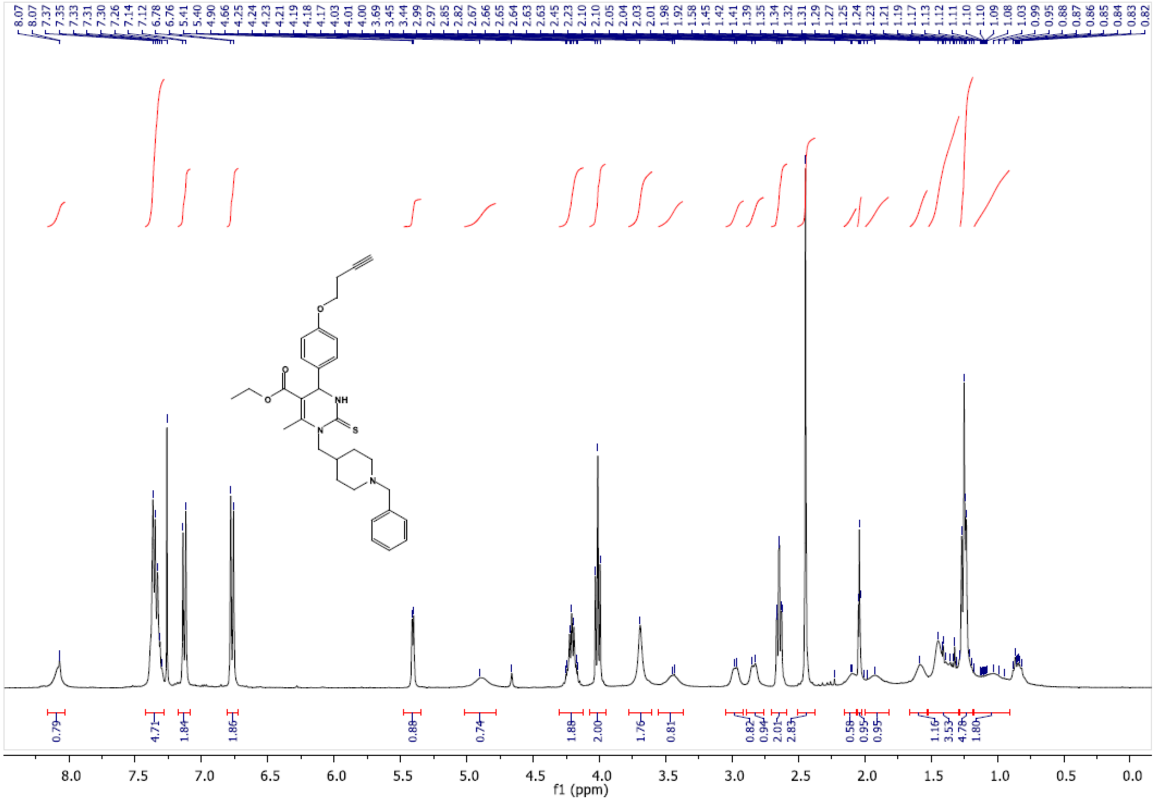

Figure S12: ^{13}C NMR spectrum of compound 5c

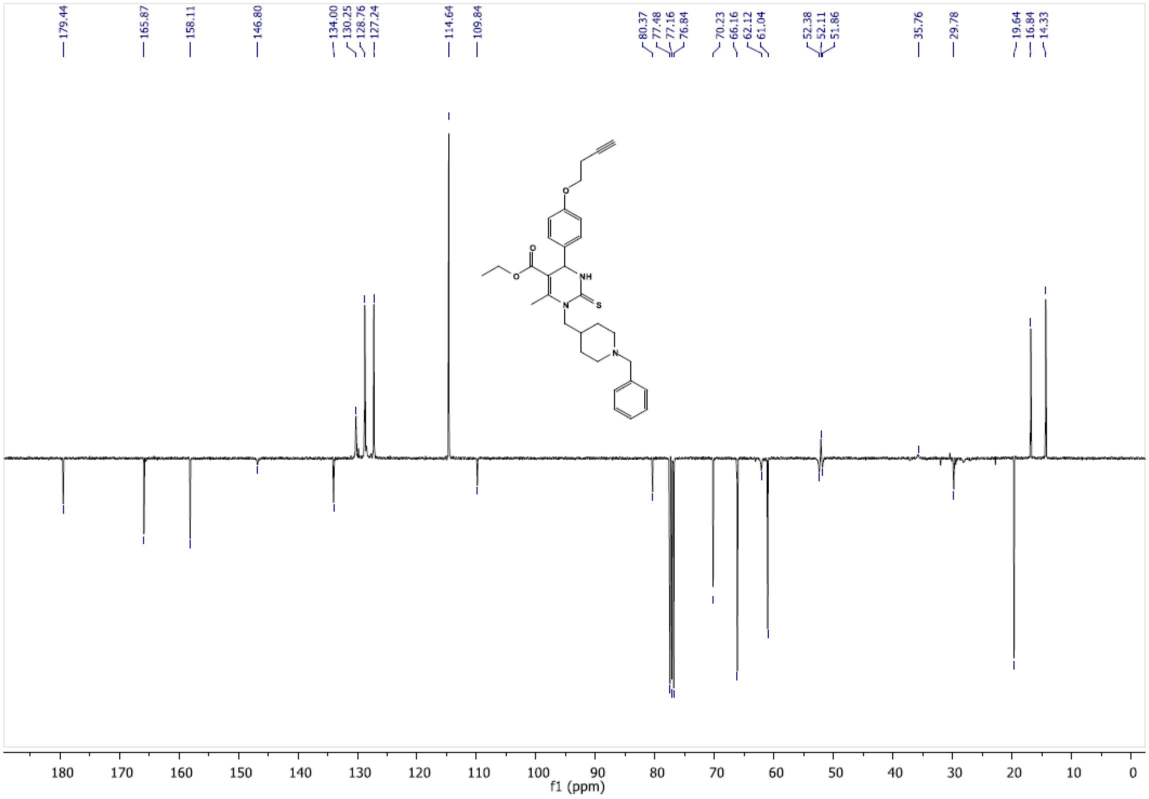

Figure S13: ^{1}H NMR spectrum of compound 4d

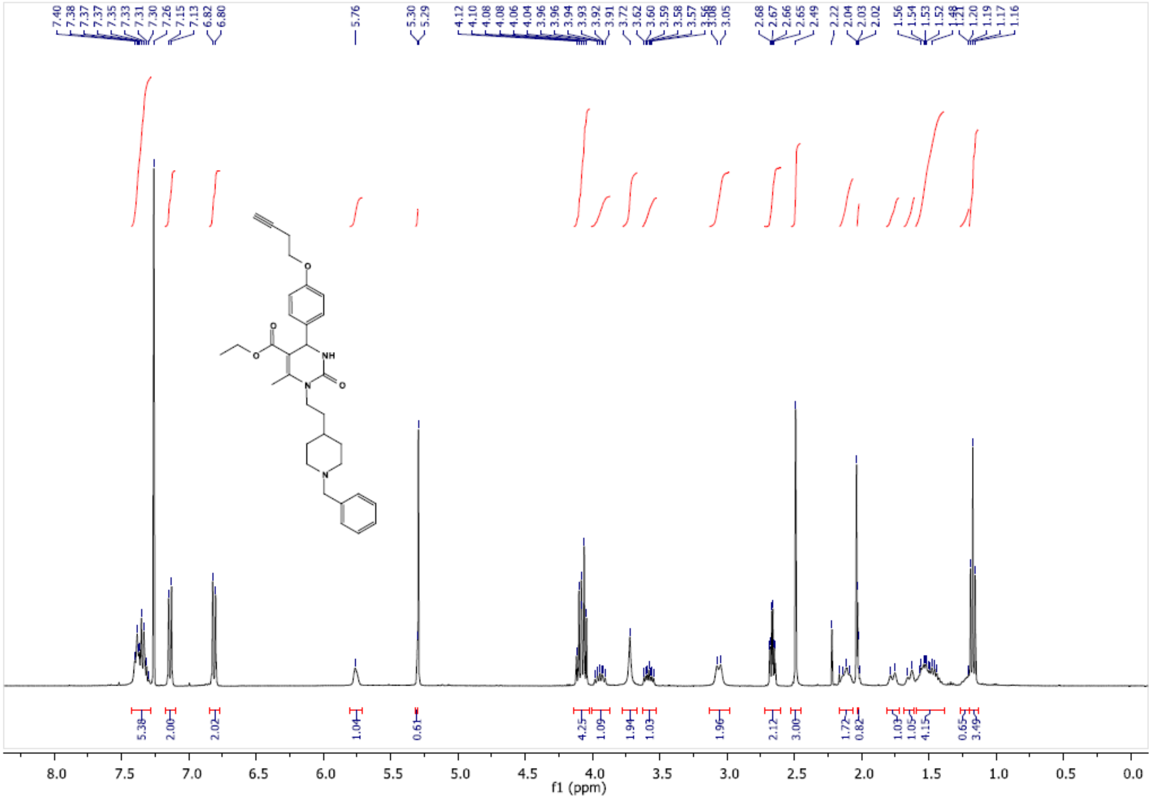

Figure S14: ^{13}C NMR spectrum of compound 4d

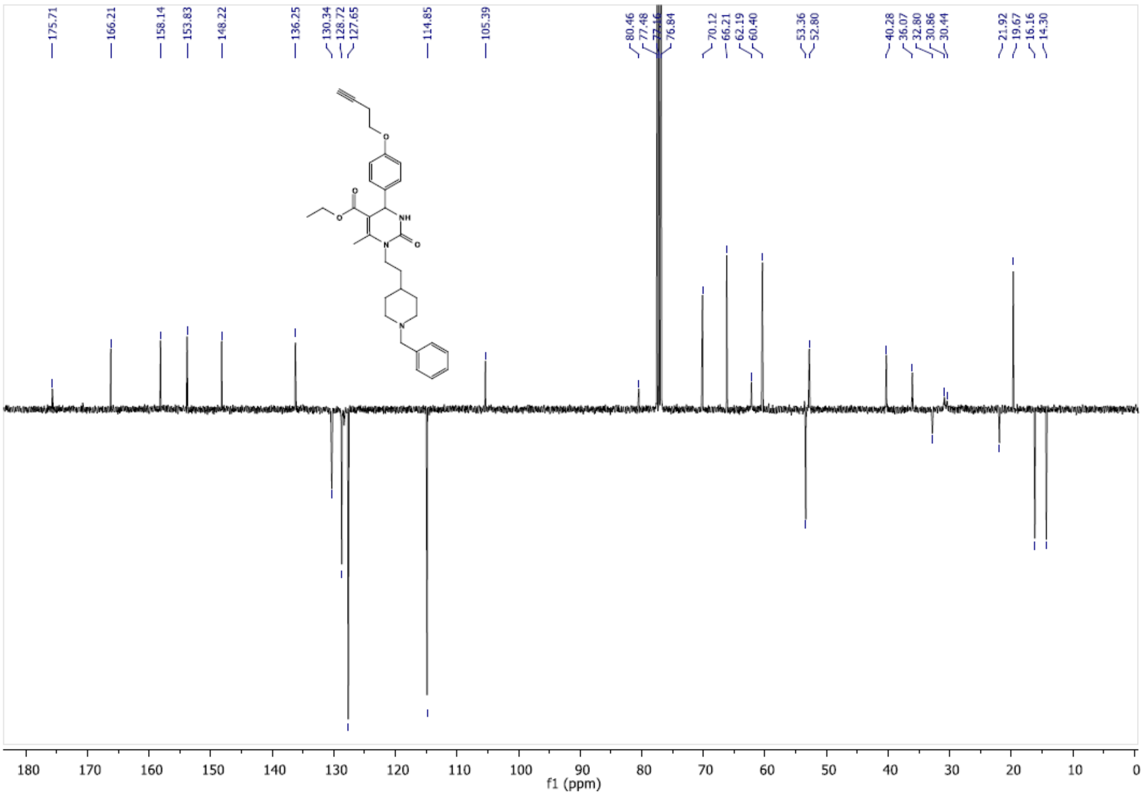

Figure S15: ^{1}H NMR spectrum of compound 5d

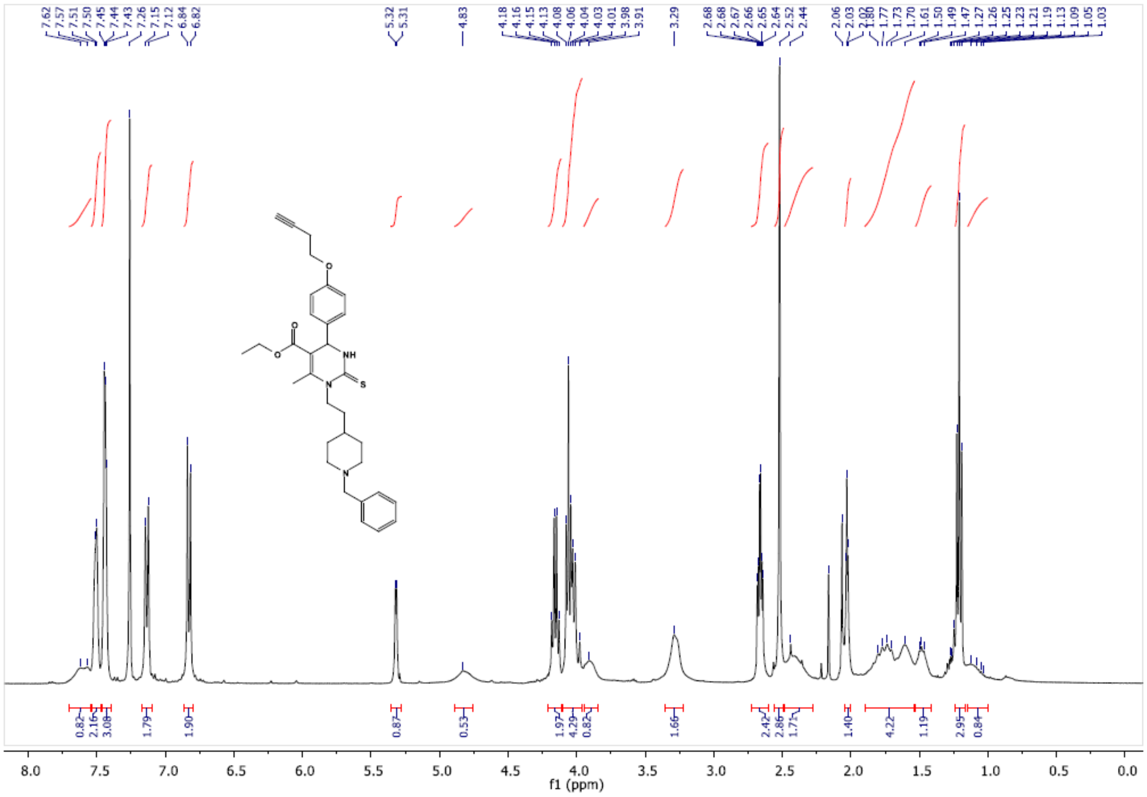

Figure S16: ^{13}C NMR spectrum of compound 5d

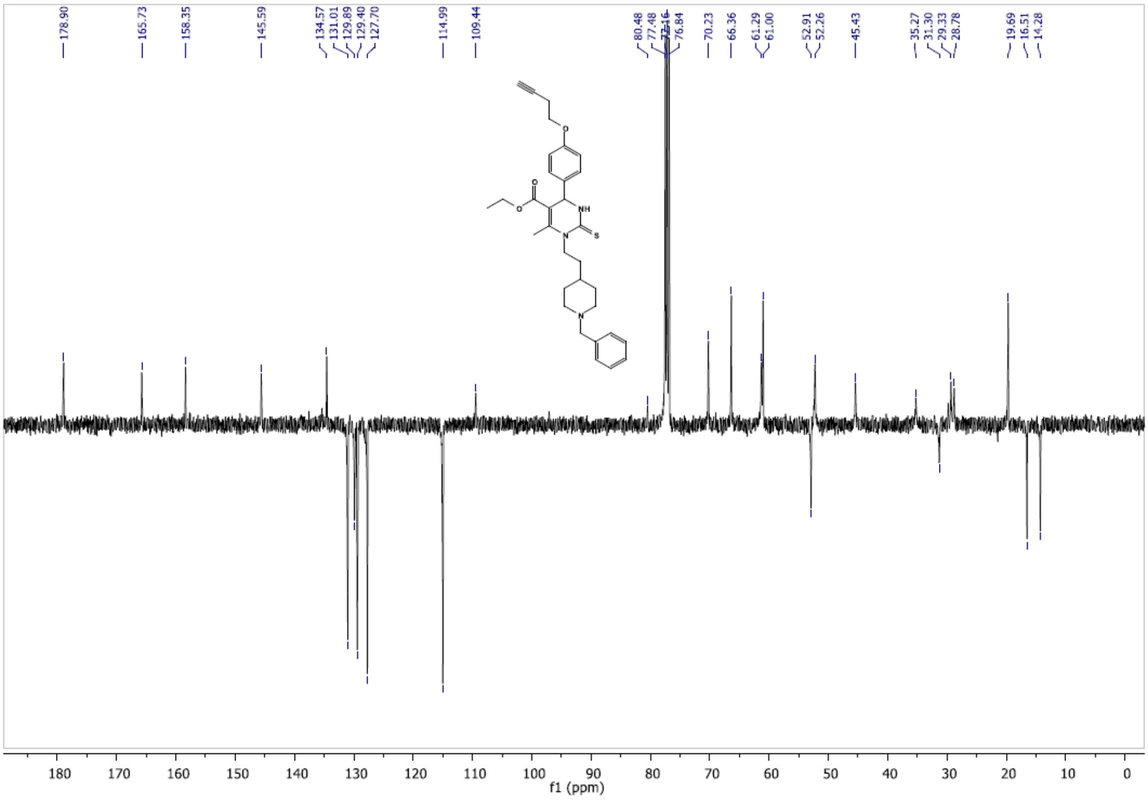

Supplement: Supplementary file 1 [file molecules-28-00071-s001.zip › molecules-2092647-supplementary.pdf]
